# COVID-19 Pandemic and Quality of Life among Romanian Athletes

**DOI:** 10.3390/ijerph18084065

**Published:** 2021-04-12

**Authors:** Germina-Alina Cosma, Alina Chiracu, Amalia Raluca Stepan, Marian Alexandru Cosma, Marian Costin Nanu, Florin Voinea, Khalid Walid Bibi, Cătălin Păunescu, Monoem Haddad

**Affiliations:** 1 Faculty of Physical Education and Sport, University of Craiova, 200585 Craiova, Romania; germina.cosma@edu.ucv.ro (G.-A.C.); amalia.raluca.stepan@gmail.com (A.R.S.); alexandrucosma_78@yahoo.com (M.A.C.); costi_21nanu@yahoo.com (M.C.N.); 2 Faculty of Psychology and Science Education, University of Bucharest, 050663 Bucharest, Romania; alina.chiracu@drd.unibuc.ro; 3 Faculty of Physical Education and Sport, University Ovidius of Constanța, 900470 Constanța, Romania; voineaflorin09@yahoo.com; 4 Physical Education Department, College of Education, Qatar University, Doha 2713, Qatar; bibi@qu.edu.qa; 5 Physical Education Department, University of Medicine and Pharmacy Carol Davila, 020021 Bucharest, Romania; catalin.paunescu@umfcd.ro

**Keywords:** COVID-19, athletes, neuroticism, vulnerability, quality of life

## Abstract

The aim of this study was to analyze athletes’ quality of life during the COVID-19 pandemic. The study involved 249 athletes between 15 and 35 of age, M = 21.22, SD = 5.12. The sample was composed of eight Olympic Games medalists, three European medalists, 67 international medalists, and 63 national medalists. The instruments used were: (1) COVID-19 Anxiety Scale, (2) Athlete Quality of Life Scale, (3) Impact of Pandemic on Athletes Questionnaire, and (4) International Personality Item Pool (IPIP Anxiety, Depression, and Vulnerability Scales). The results indicate significant differences in COVID-19 anxiety depending on the sport practiced, F (9239) = 3.81, *p* < 0.01, showing that there were significant differences between sports. The negative impact of the COVID-19 pandemic mediates the relationship between trait anxiety and the athletes’ quality of life. The percentage of mediation was 33.9%, and the indirect effect was −0.11, CI 95% (−0.18, −0.03), Z = −2.82, *p* < 0.01. Trait anxiety has an increasing effect on the intensity of the negative impact of the COVID-19 pandemic, 0.23, CI 95% (.10, 0.35), Z = 3.56, *p* < 0.01, and the negative impact of the COVID-19 pandemic has a decreasing effect on quality of life, −0.47, CI 95% (−0.67, −0.27), Z = −4.62, *p* < 0.01. Gender and age did not moderate the relationship between the negative impact of COVID-19 and athletes’ quality of life. The results of the study highlighted the impact that social isolation and quarantine have on athletes’ affective well-being.

## 1. Introduction

The COVID-19 pandemic has brought significant changes in the lives of most people around the world. Like other pandemics throughout history, COVID-19 has a profound effect on people’s anxiety, distress, fear, uncertainty [1]. Athletes represent a unique subset of the greater population because the vast majority cannot train in isolation.

The COVID-19 pandemic restricted athletes’ physical activity and training regardless of the type of sport performed. On the one hand, interruptions to the nature and regularity of the training sessions could have detrimental effects on the athletes’ physical condition. On the other hand, the cancellation of the sports competitions could have damaging effects on their social and economic life [2].

Based on negative effects reported in previous research on the COVID-19 pandemic, a decrease in the quality of athletes’ life is expected [3]. Training routines play a fundamental role in their mental health and well-being. Dramatic variations in training routines, loss of social support, or absence of leadership and external motivation can lead to high levels of stress and frustration. Limiting or eliminating group training deprives some athletes from an important mechanism to counter the social support they rely on to mitigate symptoms of depression, anxiety, or stress caused by the pandemic [2]. During this period, it is necessary to support athletes psychologically through regular online consultations and by encouraging the maintenance of minimal social interactions with family, friends, and teammates [4].

Athletes lead robust social lives. Training and competitions play a vital role in that aspect of their lives. Moreover, they participate in competitions where they are in the presence of large flux of people. Full or partial social isolation imposed by the COVID-19 pandemic deprives athletes from these two critical social settings [5]. When athletes are forced to train at home, to receive online instructions from coaches, to find ways to compensate for the absence of teammates, they may eventually experience increasing levels of emotional and affective symptoms [6].

Regardless of contamination risks and the negative impact of the imposed restrictions, athletes’ quality of life is directly affected on all levels [7]. The most well-known term that defines the quality of life is satisfaction with life, which is often considered a component of quality of life. Satisfaction with life depends on how the individual perceives his living conditions in general. Varca, et al. [8] argued that life satisfaction could be viewed as a general index of a person’s emotional or affective responses to his or her life and well-being.

Athletes’ quality of life and consequently their satisfaction with life is greatly influenced by their satisfaction with their professional achievements [9], to which is added the effort they make in achieving performance, longevity in sports careers, quality of relationships with teammates, and the general level of happiness.

In their attempt to define the athletes’ satisfaction with life, Whittal and Orlick [10] identified six constituting components: the sport practiced, the type of training, the coach, teammates, opponents, and the level of performance. Riemer and Chelladurai [11] suggested that athletes’ satisfaction occurs when their sporting experiences meet their needs, so they defined athletes’ satisfaction with lives as a positive emotional state resulting from a complex assessment of structural processes and outcomes of sports experiences. Furthermore, this positive state is mediated by the discrepancies between what they want and what they manage to achieve. The World Health Organization describes the quality of life as people’s perception of their situation within the culture and their own value system in relation to their goals, standards, expectations, and ideas [12].

The Big Five personality model describes through its Neuroticism component six aspects of emotional instability: anxiety, impulsivity, depression, low self-consciousness, irritability, and vulnerability. Trait anxiety is part of the personality construct and represents an acquired tendency or disposition that influences behavior [13]. This predisposes the person to have a stable tendency to experience and report negative emotions and anxiety in most situations. People react to these situations with high levels of anxiety, disproportionate in intensity and magnitude to the real danger [14]. Trait anxiety can be analyzed as a distinct personality trait or within the neuroticism dimension, characterized by experiencing negative emotions such as fear, guilt, and frustration [15].

The associations between trait anxiety and quality of life are highlighted in numerous studies. The characteristics of anxiety are considered significant predictors of a low level of well-being and quality of life, both psychosocially and cognitively [16,17,18]. Frisch [19] showed a negative association between trait anxiety and quality of life among young people with and without mental disorders. Millon and Davis [20] found that trait anxiety is a negative predictor of quality of life due to lack of joy, negativity, and lack of satisfaction with life.

Trait depression is also part of the neuroticism personality factor and refers to permanence of sadness, negative affect, lack of hope, and planning for the future. Kato, et al. [21] developed an instrument to measure trait depression, highlighting its three dimensions: avoiding social roles, low self-esteem, and worry. People with high levels of trait depression tend to see life in gloomy light, misinterpret the events they are exposed to, and be pessimistic. Chambers et al. have shown that trait anxiety and trait depression are determinants of anxiety and depression states and disorders, leading to worsening and chronicity of their symptoms [22].

Vulnerability as a dimension of neuroticism refers to people’s tendency to encounter problems in coping with stress and a feeling of helpless in the face of life’s challenges. The effects of a high level of vulnerability are reflected on health and implicitly on quality of life [23].

Overall, people with high levels of neuroticism more frequently report medically unfounded somatic problems and have a catastrophic view of the symptoms they experience [24]. Numerous studies have shown that neuroticism is also associated with genuine and real health problems [25,26,27,28]. Because neuroticism is associated with physical and mental health problems, it is implicitly correlated with the comorbidity between them, implying a greater need for medical services.

According to the diathesis-stress model of psychopathology, each individual possesses an inherent vulnerability to develop a certain disorder. The development of the disorder can be triggered by stressors from the environment, but the level of stress experienced in contact with environmental factors is closely related to the pre-existing level of vulnerability of the individual. Kendler, et al. [29] showed that genetic risk factors make the individual vulnerable to adverse events. Compared to the less vulnerable individuals, they are more likely to develop depression and anxiety due to exposure to stressful situations.

Psychological distress is usually associated with the gender and age of athletes. Thus, according to Lee [30], both adaptation and reaction to events with high stress potential may have different manifestations in men than in women or in young athletes compared to adults. Although young people are less vulnerable to COVID-19, they are more likely to have mental disorders due to its consequences, such as isolation and reduced social contact [31]. We can therefore assume that gender and age can be moderating variables in the relationship between the negative impact of the COVID-19 pandemic and the quality of life of athletes.

Although internationally there are already several studies on the impact of the COVID-19 pandemic on the mental health of athletes [32,33,34], as far as we know, this is the first study on the quality of life of Romanian athletes during the COVID-19 pandemic.

Taking into account the above, we assume that overall vulnerability conferred by high levels of neuroticism leads to a marked decrease in quality of life and that against a background of anxious, depressed or vulnerable personality, the negative impact of COVID-19 pandemic will cause a more pronounced decrease in athletes’ quality of life.

To test these assumptions, we established a series of hypotheses:

**H1.**  *Athletes who practice individual sports have higher levels of COVID-19 anxiety than those who practice team sports*.

**H2.**  *Athletes who practice individual sports feel more strongly the negative impact of the COVID-19 pandemic than athletes who practice team sports*.

**H3.**  *The negative impact of the COVID-19 pandemic mediates the relationship between trait anxiety and athletes’ quality of life*.

**H4.**  *The negative impact of the COVID-19 pandemic mediates the relationship between trait depression and athletes’ quality of life*.

**H5.**  *The negative impact of the COVID-19 pandemic mediates the relationship between trait vulnerability and athletes’ quality of life*.

**H6.**  *Athletes’ gender moderates the relationship between the negative impact of COVID-19 pandemic and athletes’ quality of life*.

**H7.**  *Athletes’ age moderates the relationship between the negative impact of COVID-19 pandemic and athletes’ quality of life*.

The purpose of this study was to identify changes that occurred in athletes’ personal and professional life because of the introduction of physical distancing measures during the COVID-19 pandemic. We analyzed the relationship among COVID-19 anxiety, individual vulnerability factors, such as certain personality traits related to neuroticism (anxiety, depression, and vulnerability), sociodemographic characteristics (gender and age), the negative impact of COVID-19 pandemic and the self-reported quality of life.

## 2. Materials and Methods

### 2.1. Participants

The study involved 249 Romanian athletes aged between 15 and 35 years, M = 21.22, SD = 5.12. Among them, 105 were males (42%) and 144 were females (58%), 27 athletes were high school students (11%), 10 were university students (4%), and 212 were either high school students or university students enrolled in certain sport clubs (86%). The sample includes eight Olympic Games medalists (4%), three European medalists (1%), 67 international medalists (27%), 63 national medalists (25%), and 108 non-medalists (43%). One hundred and seventeen athletes participated in national competitions (47%), 10 in European competitions (4%), 111 in international competitions (45%), and 11 in Olympic Games (4%). All participants live in Romania.

### 2.2. Procedure

Data were collected during quarantine when sports activities were completely banned, 25 March 2020–25 April 2020. The athletes were contacted online through their teachers or coaches, given a brief presentation of the research, and invited to participate. All participants in the study became aware of the processing of personal data and agreed to the consent form. Our research respects the international ethical recommendations regarding the absolute confidentiality of the data collected in the study, as well as the anonymity and security of the participants. After accepting participation, the questionnaire was administered cross-sectionally using Google Form. The link was disseminated through email and social networks. The first section of the form included the informed consent and the agreement for the processing of personal data. Teachers and coaches obtained parental consent for participants under 18 years of age (on their correspondence online group). The only inclusion criteria was to belong to an organized sport. The procedures were clearly explained, and participants could interrupt or quit the survey at any point. All subjects participated voluntarily and there were not any incentives or rewards for partaking in this study. The duration of completing the questionnaires was about 15 min. Ethics approval was obtained from the Ethic Commission of Craiova Faculty of Physical Education and Sport in conformity with the principles embodied in the Declaration of Helsinki. The present study has a cross-sectional, differential, and correlational design. Data and statistical analyses were performed through SPSS 24 (IBM, Armonk, NY, USA). The medmod module of Jamovi [35] was used to explore whether a mediating variable can explain the relationship between two variables.

### 2.3. Instruments

Sociodemographic data were collected on biological sex, age, education, and performed sport.

COVID-19 anxiety was measured with COVID-19 Anxiety Scale. This scale was developed for this study and aims to capture the extent to which the pandemic is a cause for athletes’ concern. The scale is composed of four items, and the scores are given on a five-step Likert scale, where 1 indicates a very low effect and 5 indicates a very high effect. Examples of items: “To what extent do you think the COVID-19 pandemic will affect your area of residence?” or “To what extent do you think it is possible to get COVID-19?”.

The athletes’ quality of life was measured with The Athlete Quality of Life Scale [36]. The scale includes 15 items formulated as responses to the question: “To what extent are you satisfied with the following aspects of your life?” (physical health, leisure time, social life, physical condition, relationship with the coach, etc.). The answers are given on a six-step Likert scale where 0 indicates very dissatisfied and 5 indicates very satisfied. The scale was translated by two psychologists proficient in English and back-translated by two licensed translators. After making the necessary corrections to maintain the meaning of the items, it was entered in Google form.

The negative impact of COVID-19 pandemic was measured with The Impact of Pandemic on Athletes Questionnaire. The instrument was developed by the authors of this study to capture the immediate effects of the pandemic on the personal and professional lives of athletes. The questionnaire includes 10 items, and the answers are provided on a five-step Likert scale where 1 indicates a small extent and 5 indicates a large extent. Examples of items: “The isolation period affected my training program” or “I have always received online advice from my coach” (reverse scoring).

Anxiety, depression, and vulnerability as personality traits adjacent to neuroticism were measured with three subscales of the International Personality Item Pool, translated into Romanian [37]. Each subscale contains 10 items, and the answers are given on a five-step Likert scale, where 1 - total disagreement and 5 - total agreement. Examples of items: for anxiety: “I’m afraid of many things”, for depression: “I feel that my life lacks direction” and for vulnerability: “I become overwhelmed by events”. The questionnaires included in this study are presented in the Supplementary Data of the article.

## 3. Results

Descriptive statistics and correlations among variables are reported in Table 1.

Hypothesis testing

**H1.**  *Athletes who practice individual sports have higher levels of covid-19 anxiety than those who practice team sports*.

**Figure 1:**
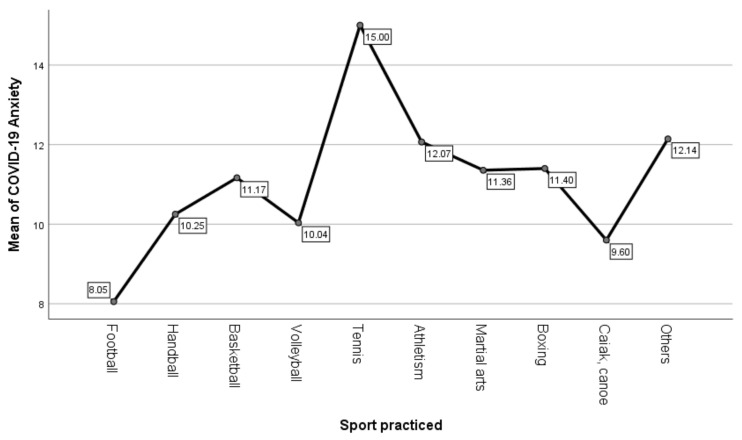
Mean scores for COVID-19 anxiety depending on the sport practiced.

Differences in COVID-19 anxiety between athletes who practice individual sports and those who practice team sports are presented in Table 2. A one-way ANOVA analysis of variance was performed for all ten sports categories. Graphic representation of mean scores is presented in Figure 1.

It was observed that there are significant differences in COVID-19 anxiety depending on the sport practiced, F (9239) = 3.81, *p* < 0.01. The highest scores were observed in athletes who practice tennis (M = 15.00, SD = 4.58), followed by athletes who practice other sports—fencing, gymnastics (M = 12.14, SD = 5.43) and track and field (M = 12.07, SD = 3.58), and the lowest scores were observed in athletes who practice football (M = 8.05, SD = 2.99) and in those who practice kayak-canoe (M = 9.60, SD = 2.79).

**H2.**  *Athletes who practice individual sports feel more strongly the negative impact of the covid-19 pandemic than athletes who practice team sports*.

**Figure 2:**
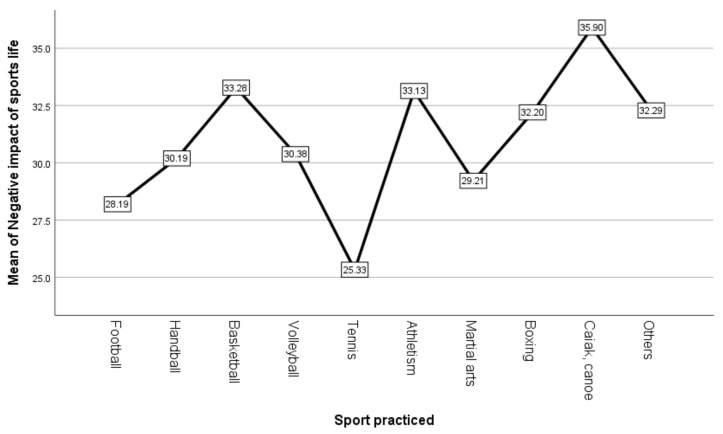
Mean scores for negative impact depending on the sport practiced.

Differences regarding negative impact of COVID-19 between athletes who practice individual sports and those who practice team sports are presented in Table 3. A one-way ANOVA analysis of variance was performed for all ten sports categories. Graphic representation of mean scores is presented in Figure 2.

Regarding the negative impact of the COVID-19 pandemic, it was observed that there were significant differences depending on the sport practiced, F (9239) = 2.24, p < 0.05. The highest scores were observed in athletes who practice kayak-canoe (M = 35.90, SD = 4.46), in those who practice basketball (M = 33.28, SD = 6.23) and track and field (M = 33.13, SD = 5.99), and the lowest scores were observed in athletes who practice tennis (M = 25.33, SD = 2.31) and in those who practice football (M = 28.19, SD = 7.69).

**H3.**  *The negative impact of the covid-19 pandemic mediates the relationship between trait anxiety and athletes’ quality of life*.

A mediation analysis was performed with trait anxiety as predictor, negative impact of COVID-19 as mediator and athletes’ quality of life as dependent variable. Mediation estimates are presented in Table 4.

The negative impact of the COVID-19 pandemic mediates the relationship between trait anxiety and the athletes’ quality of life, the percentage of mediation was 33.9% and the indirect effect was −0.11, CI 95% (−0.18, −0.03), Z = −2.82, *p* < 0.01. Trait anxiety has an increasing effect on the intensity of the negative impact of the COVID-19 pandemic, 0.23, CI 95% (0.10, 0.35), Z = 3.56, *p* < 0.01, and the negative impact of the COVID-19 pandemic has a decreasing effect on quality of life, −0.47, CI 95% (−0.67, −0.27), Z = −4.62, *p* < 0.01.

**H4.**  *The negative impact of the covid-19 pandemic mediates the relationship between trait depression and athletes’ quality of life*.

A second mediation analysis was performed with trait depression as predictor, negative impact of COVID-19 as mediator and athletes’ quality of life as dependent variable. Mediation estimates are presented in Table 5.

Also, the negative impact of the COVID-19 pandemic mediates the relationship between trait depression and the athletes’ quality of life, the percentage of mediation was 26.4% and the indirect effect was −0.09, CI 95% (−0.16, -0.03), Z = −2.75, *p* < 0.01. Trait depression has an increasing effect on the intensity of the negative impact of the COVID-19 pandemic, 0.20, CI 95% (0.09, 0.32), Z = 3.48, *p* < 0.01, and the negative impact of the COVID-19 pandemic has a decreasing effect on quality of life, −0.46, CI 95% (−0.66, −0.26), Z = −4.51, *p* < 0.01.

**H5.**  *The negative impact of the covid-19 pandemic mediates the relationship between trait vulnerability and athletes’ quality of life*.

A third mediation analysis was performed with trait vulnerability as predictor, negative impact of COVID-19 as mediator and athletes’ quality of life as dependent variable. Mediation estimates are presented in Table 6.

It was also observed that the negative impact of the COVID-19 pandemic mediates the relationship between trait vulnerability and the athletes’ quality of life, the percentage of mediation was 23.6% and the indirect effect was −0.09, CI 95% (−0.16, -0.03), Z = −2.87, *p* < 0.01. Trait vulnerability has an increasing effect on the intensity of the negative impact of the COVID-19 pandemic, 0.22, CI 95% (0.11, 0.33), Z = 3.83, *p* < 0.01, and the negative impact of the COVID-19 pandemic has a decreasing effect on quality of life, −0.44, CI 95% (−0.64, −0.24), Z = −4.33, *p* < 0.01. Considering these results, we can conclude that our assumptions were supported by data.

**H6.**  *Athletes’ gender moderates the relationship between the negative impact of COVID-19 pandemic and athletes’ quality of life*.

A series of three moderation analyses were performed with trait anxiety, trait depression and trait vulnerability as predictors, gender as moderator, and athletes’ quality of life as dependent variable. Moderations’ estimates are presented in Table 7.

**H7.**  *Athletes’ age moderates the relationship between the negative impact of COVID-19 pandemic and athletes’ quality of life*.

A second series of three moderation analyses were performed with trait anxiety, trait depression and trait vulnerability as predictors, age as moderator and athletes’ quality of life as dependent variable. Moderations’ estimates are presented in Table 8.

It can be seen that neither gender nor age moderate the relationship between trait anxiety/trait depression/trait vulnerability and the athletes’ quality of life.

## 4. Discussion

Through hypotheses H1 and H2 we aimed to determine if there are differences regarding COVID-19 anxiety and negative impact of the pandemic depending on the sport practiced. The results showed that there were significant differences between sports. However, there were no differences on this variable between athletes who participate in individual vs. team sports.

These results can be attributed to the fact that their training normally takes place outdoors and requires specific deployment conditions, conditions which are hindered due to the pandemic and restrictions on free movement. The training of these athletes occurs more heavily in restrictive environments such as their own home and moreover, the lack of teammates makes them feel truly socially isolated and unable to maintain good physical condition. These results support our assumptions that individually trained athletes experience more painful the imposed social isolation and are considered more vulnerable to SARS-COV 2 contamination, probably due to the lack of social support from teammates.

Regarding the impact of COVID-19 anxiety on the quality of life of athletes, previous studies have obtained heterogeneous results. While some have shown that athletes who practice individual sports have a higher decrease of mental health than those who practice team sports [38,39], others have found the opposite results [32]. It is possible that these differences are also caused by the nature of the training, not only by the presence or absence of teammates. Those athletes who manage to train at home or in specially designed areas suffer less than those who do not have this possibility, regardless of the sport practiced.

Hypotheses H3, H4 and H5 showed that the vulnerability background of the participants through high levels of neuroticism (anxiety, depression, vulnerability) has increasing effects on experiencing the negative impact of the pandemic, which leads to decreased quality of life. Against a background of vulnerability caused by certain personality traits, such as anxiety, depression or vulnerability, it seems that the negative impact of COVID-19 pandemic is more pronounced, leading to a decrease in the quality of life of athletes in all areas. It is known that critical life events have strong negative effects on the mental health but also on the physical health of the entire population [40] and especially those in situations at high risk of contamination, such as athletes.

Similar results were obtained by Unalan, et al. [41], who conducted a study on the relationships between trait anxiety and quality of life in which 276 students participated. The results showed that trait anxiety was responsible for diminishing the quality of life in terms of physical health, mental health, and the level of independence and interpersonal relationships. Keyzer-Dekker, et al. [42] showed in a longitudinal study that higher levels of trait anxiety lead to a decreased quality of life among women with and without cancer. Zautra, et al. [43], in a study based on the daily diaries of patients with rheumatoid arthritis, showed that people with high levels of neuroticism experienced greater negative affect in the days when they faced negative life events. Longitudinal studies have also shown that people with high neuroticism are more likely to develop anxiety and depression when faced with negative life events [44,45].

Through hypotheses H6 and H7 we aimed to determine if age and gender moderate the relationship between the negative impact of COVID-19 pandemic and athletes’ quality of life. Neither variable moderates this relationship. This result may be caused by the fact that the COVID-19 pandemic is a far too strong life event with a much too specific impact, which spreads to the entire population, so that sociodemographic differences are no longer factors that have significant effects. There are not enough studies on the athletes’ quality of life of in pandemic conditions to make additional references to them.

## 5. Conclusions

The present study highlighted the impact that social isolation and quarantine has on athletes’ quality of life and affective well-being. Their lives are severely affected on different levels (personal life, professional life, teammates and coach interactions, financial status, competitions participation, training and exercise). The vulnerability conferred by personality traits is a risk factor that leads to a decrease in the athletes’ quality of life, especially in conditions of a pandemic. Anxiety, depression and vulnerability as dimensions of neuroticism increase the negative perception of the impact of the COVID-19 pandemic. Among the factors that determine the negative impact of the COVID-19 pandemic are the disruption of the training program, the lack of favorable conditions for training at home, poor communication with coaches or teammates and last but not least the absence of regular medical and psychological checks.

The impact of the COVID-19 pandemic on Romanian athletes has an immediate impact on athletes’ lives in the form of a lack of quality training, a decrease in the number of national and international competitions, poor communication between athletes and teammates, but also between athletes and coaches. To these are added the financial problems resulting from not participating in competitions, which leads to a decrease in income. Psychologically, athletes are at risk of experimenting feelings of worthlessness and inner emptiness, diminished self-efficacy and self-confidence. Physically, the lack of adequate training can lead to changes in body weight, decreased resistance to physical effort, sleep and nutrition disorders with harmful effects professionally and personally.

Given these results, there is a need to implement multifaceted programs to support the athletes through individual and group psychological counseling sessions through which they can overcome the difficult moments caused by the restriction of training and competitions. Athletes should be supported at least remotely, counseled to maintain good physical and mental condition. Coaches and psychologists must maintain constant contact with them and develop training programs adapted to the existing conditions. Athletes’ careers can be jeopardized by such events, which at the same time impacts their income level. Thus, specific measures are required to support athletes, possibly through the clubs in which they operate and until the end of the quarantine period.

The Ministry of Youth and Sports of Romania left to the decision of each sport federation the application of the plans of measures regarding the fight against the spread of the SARS Cov 2 virus, these measures aiming only at the prevention and testing part, not at all at remedial actions in order to help the athletes. The Ministry of Youth and Sports of Romania provide some regulations on the conditions necessary to be observed for access to sports facilities, for the practice of individual outdoor sports, for the practice of outdoor team sports, for the resumption of swimming activities in indoor and outdoor pools in Romania, in order to carry out sports activities in closed spaces, respectively for carrying out physical training activities in fitness and aerobics gyms, according to the Order published in the Official Monitor Part I, No. 872 /24.IX.2020 [46].

Athletes, through the competitions they participate in and the results they obtain, are practically the exponents of the country they come from, often bringing joy and emotion to all citizens. With the cessation of sports competitions, not only does their income decrease, but also the very meaning of their existence is diminished, and the possibility for them to assert themselves on international podiums disappears.

The strengths of this study are the collection of data from a large number of athletes in the lockdown period. At that time there were not enough studies to refer to. We also did not identify studies that take into account the psychological vulnerabilities of athletes. At the same time, our study can be a source of information for sports specialists so as to improve their methods of training and caring for athletes in critical life contexts. Psychological and environmental aspects were captured regarding the negative impact of COVID-19 pandemic on quality of life of athletes, thus facilitating the search for solutions to remedy the existing situation.

The limits of the study consist in the online application of the questionnaires, in the lack of equivalence of the compared groups and the use of self-reported scales. In addition, the use of two instruments unvalidated in Romanian and the absence of psychometric properties of these may be a limitation.

## Figures and Tables

**Table 1:** Means, standard deviations, reliability coefficients, and correlations among variables.

Variable		Range	M	SD	α	CA	QoL	NI	A	D	V
CA		16	10.22	3.43	0.76	1					
QoL		52	47.71	11.25	0.88	0.02	1				
NI		32	30.55	6.76	0.76	0.15 *	−0.31 **	1			
A		33	27.20	6.54	0.73	0.10	−0.18 **	0.22 **	1		
D		35	20.55	7.13	0.83	0.05	−0.22 **	0.22 **	0.49 **	1	
V		39	21.77	7.37	0.87	0.12	−0.26 **	0.24 **	0.56 **	0.63 **	1

*. *p* < 0.05, **. *p* < 0.01. CA = COVID-19 anxiety, QoL—Quality of life, NI—Negative impact of pandemic on athletes, A—Anxiety trait, D—Depression trait, V—Vulnerability trait.

**Table 2:** One-way ANOVA for COVID-19 anxiety depending on the sport practiced.

	Sum of Squares	df	Mean Square	F	Sig.
Between Groups	366.18	9	40.69	3.81	0.00
Within Groups	2550.68	239	10.67		
Total	2916.85	248			

**Table 3:** One-way ANOVA for negative impact of COVID-19 pandemic depending on the sport practiced.

	Sum of Squares	df	Mean Square	F	Sig.
Between Groups	880.37	9	97.82	2.24	0.02
Within Groups	10445.25	239	43.70		
Total	11325.62	248			

**Table 4:** Mediation analysis for negative impact of COVID-19 pandemic in the relationship between trait anxiety and quality of life.

				95% CI				
Effect	Label	Estimate	SE	Lower	Upper	Z	*p*	% Mediation
Indirect	a × b	−0.11	0.04	−0.18	−0.03	−2.82	0.01	33.9
Direct	c	−0.21	0.11	−0.42	−0.01	−1.99	0.05	66.1
Total	c + a × b	−0.32	0.11	−0.53	−0.11	−2.95	0.00	100.0

**Table 5:** Mediation analysis for negative impact of COVID-19 pandemic in the relationship between trait depression and quality of life.

				95% CI				
Effect	Label	Estimate	SE	Lower	Upper	Z	*p*	% Mediation
Indirect	a × b	−0.09	0.03	−0.16	−0.03	−2.75	0.01	26.4
Direct	c	−0.26	0.09	−0.45	−0.07	−2.71	0.01	73.6
Total	c + a × b	−0.35	0.10	−0.55	−0.16	−3.62	< 0.001	100.0

**Table 6:** Mediation analysis for negative impact of COVID-19 pandemic in the relationship between trait vulnerability and quality of life.

				95% CI				
Effect	Label	Estimate	SE	Lower	Upper	Z	*p*	% Mediation
Indirect	a × b	−0.09	0.03	−0.16	−0.03	−2.87	0.00	23.6
Direct	c	−0.31	0.09	−0.49	−0.12	−3.31	< 0.001	76.4
Total	c + a × b	−0.40	0.09	−0.58	−0.22	−4.29	< 0.001	100.0

**Table 7:** Moderation analysis for gender in the relationship between trait anxiety/trait depression/trait vulnerability and quality of life.

95% CI							
Predictor	Moderator	Estimate	SE	Lower	Upper	Z	*p*
Trait anxiety	Gender	−0.32	0.22	−0.74	0.10	−1.48	0.14
Trait depression	Gender	−0.24	0.21	−0.64	0.17	−1.15	0.25
Trait vulnerability	Gender	−0.12	0.21	−0.53	0.30	−0.55	0.58

**Table 8:** Moderation analysis for age in the relationship between trait anxiety/trait depression/trait vulnerability and quality of life.

95% CI							
Predictor	Moderator	Estimate	SE	Lower	Upper	Z	*p*
Trait anxiety	Age	−0.01	0.02	−0.05	0.03	−0.61	0.54
Trait depression	Age	−0.01	0.02	−0.05	0.04	−0.31	0.76
Trait vulnerability	Age	0.01	0.02	−0.03	0.04	0.46	0.65

## Data Availability

The raw data supporting the conclusions of this article will be made available by the authors, without undue reservation.

## Supplementary Materials

The Supplementary Materials are available online at https://www.mdpi.com/article/10.3390/ijerph18084065/s1.
