# Supplementary material for: COVID-19 Pandemic and Quality of Life among Romanian Athletes
Source: Int J Environ Res Public Health. 2021 Apr 12;18(8):4065. doi: 10.3390/ijerph18084065 (PMC8069478; doi:10.3390/ijerph18084065)
Supplement: Supplementary file 1 [file ijerph-18-04065-s001.pdf]

Supplementary Data

COVID-19 Anxiety items

1. To what extent are you concerned about COVID-19?

2. To what extent do you consider that the COVID-19 pandemic will affect your area of residence?

3. To what extent do you think it is possible to get COVID-19?

4. To what extent do you think it is possible for a loved one to get COVID-19?

Negative impact of COVID-19 items

1. The isolation period affected my training program

2. I managed to train in optimal conditions from home (R)

3. I encountered problems in improvising the equipment and installations necessary in carrying out the training

4. I have always received advice from the coach (R)

5. I handled the online training, supervised by the coach or the physical trainer (R)

6. I received psychological counseling from specialists (R)

7. I worked harder than in a normal training period

8. I kept in touch with my teammates (R)

9. I am in optimal sports shape (R)

10. When I get out of isolation, I will be able to resume my training in the same way as before March 2020 (R)

Athlete Life Quality Scale

Using the scale below, indicate how satisfied you are with the various aspects of your life listed.

1=Very Dissatisfied 2=Dissatisfied 3=Slightly Dissatisfied 4=Neutral/Undecided 5=Slightly Satisfied 6=Satisfied 7=Very Satisfied

1. Your own physical health

2. The amount of free/recovery time you have away from your sport

3. Your relationships with family members

4. Your relationships with friends

5. Your social life

6. Your relationships with your coaches
7. Your relationships with your teammates
8. Your level of physical condition
9. Your athletic performance
10. Your role on your team
11. Your financial situation
12. Your spiritual health
13. Your mental health
14. Your relationship with boyfriend/girlfriend, spouse, etc.
15. Your life as a whole
